# Treatment-Resistant Depression in Primary Care Across Canada

**DOI:** 10.1177/070674371405900702

**Published:** 2014-07

**Authors:** Sakina J Rizvi, Etienne Grima, Mary Tan, Susan Rotzinger, Peter Lin, Roger S McIntyre, Sidney H Kennedy

**Affiliations:** 1 Student, Departments of Pharmaceutical Sciences and Neuroscience, University of Toronto, Toronto, Ontario; Clinical Research Coordinator, Department of Psychiatry, University Health Network, Toronto, Ontario.; 2 Chief Operating Officer and Chief Financial Officer, Canadian Heart Research Centre, Toronto, Ontario.; 3 Statistician, Canadian Heart Research Centre, Toronto, Ontario.; 4 Project Manager, Department of Psychiatry, University Health Network, Toronto, Ontario.; 5 Director of Primary Care Initiatives, Canadian Heart Research Centre, Toronto, Ontario.; 6 Psychiatrist, Department of Psychiatry, University Health Network, Toronto, Ontario; Professor, Department of Psychiatry, University of Toronto, Toronto, Ontario; Professsor, Department of Pharmacology, University of Toronto, Toronto, Ontario.; 7 Psychiatrist, Department of Psychiatry, University Health Network, Toronto, Ontario; Professor, Department of Psychiatry, University of Toronto, Toronto, Ontario; Professor, Institute of Medical Sciences, University of Toronto, toronto, Ontario.

**Keywords:** treatment-resistant depression, prevalence, risk factors, primary care

## Abstract

**Objective:**

Treatment-resistant depression (TRD) represents a considerable global health concern. The goal of the InSight study was to investigate the prevalence of TRD and to evaluate its clinical characterization and management, compared with nonresistant depression, in primary care centres.

**Methods:**

Physicians completed a case report on a consecutive series of patients with major depressive disorder (*n* = 1212), which captured patient demographics and comorbidity, as well as current and past medication.

**Results:**

Using failure to respond to at least 2 antidepressants (ADs) from different classes as the definition of TRD, the overall prevalence was 21.7%. There were no differences in prevalence between men and women or among ethnicities. Patients with TRD had longer episode duration, were more likely to receive polypharmacy (for example, psychotropic, lipid-lowering, and antiinflammatory agents), and reported more AD related side effects. Higher rates of disability and comorbidity (axes I to III) were associated with treatment resistance. Obesity and being overweight were also associated with treatment resistance. While the selection and sequencing of pharmacotherapy by family physicians in this sample was in line with recommendations from evidence-based treatment guidelines, the wait time to make a change in treatment was 6 to 8 weeks in both groups, which exceeds guideline recommendations.

**Conclusions:**

These real-world data demonstrate the high prevalence of TRD in primary care settings, and underscore the substantial burden of illness associated with TRD.

## Abstract (fr)

**Objectif:**

La dépression réfractaire au traitement (DRT) représente un problème de santé considérable sur le plan mondial. Le but de l’étude InSight était de rechercher la prévalence de la DRT et d’en évaluer la caractérisation et la gestion cliniques, comparativement à la dépression non réfractaire, dans les centres de soins primaires.

**Méthodes:**

Les médecins ont procédé à une étude de cas sur une série consécutive de patients souffrant de trouble dépressif majeur (*n* = 1212), qui comprenait les données démographiques et les comorbidités des patients, ainsi que leurs médicaments présents et passés.

**Résultats:**

En utilisant la non réponse à au moins 2 antidépresseurs (AD) de différentes classes comme définition de la DRT, la prévalence globale était de 21,7 %. Il n’y avait pas de différence de prévalence entre hommes et femmes ou entre groupes ethniques. Les patients souffrant de DRT avaient des épisodes de plus longue durée, étaient plus susceptibles de recevoir une polypharmacie (par exemple, des agents psychotropes, des hypolipidémiants, et antiinflammatoires), et déclaraient plus d’effets secondaires liés aux AD. Des taux élevés d’invalidité et de comorbidité (axes I à III) étaient associés à la résistance au traitement. L’obésité et l’embonpoint étaient aussi associés à la résistance au traitement. Même si la sélection et la séquence de la pharmacothérapie par les médecins de famille dans cet échantillon étaient conformes aux recommandations des lignes directrices basées sur les données probantes du traitement, le temps d’attente pour effectuer un changement de traitement était de 6 à 8 semaines dans les 2 groupes, ce qui excède les recommandations des lignes directrices.

**Conclusions:**

Ces données en milieu réel démontrent la prévalence élevée de la DRT dans les soins de première ligne, et soulignent le fardeau substantiel de la maladie associé à la DRT.

Major depressive disorder is a disabling condition that results in significant economic and social burden.^1^–^3^ Much of this burden can be attributed to TRD,^4^ which is associated with a 40% to 50% increase in direct and indirect medical care costs, compared with nonresistant depression.^5^,^6^

Although there is no consensus on the definition of TRD, failure to respond to 2 or more adequate trials from different classes of ADs is the minimum requirement.^7^,^8^ The difficulty in defining TRD partly reflects the difficulty in obtaining an accurate medication history (adequacy of dose and duration for each trial), as well as incorporating new treatment strategies in the definition, such as augmentation and combination treatments. In the large STAR*D trial, the estimate of TRD, based on a failed response to at least 2 ADs, was about 30%.^9^ As these patients were recruited for treatment from primary care and psychiatric clinics, it is unclear whether this accurately reflects prevalence rates in the community. To our knowledge, no study has been reported that primarily evaluates the prevalence, determinants, and associated features of TRD within general practice.

By its very nature, TRD represents a chronic and complex illness that requires long-term management from health care professionals, usually in the form of multiple medications.^10^ This requires knowledge of adequate medication dose and duration, as well as potential drug interactions. Atypical APs are increasingly used as augmentation agents, owing to demonstrated efficacy in TRD samples.^11^–^14^ Other strategies for TRD include AD combination trials,^10^ as well as approved and experimental neurostimulation therapies, including electroconvulsive therapy, vagus nerve stimulation, repetitive transcranial magnetic stimulation, and deep brain stimulation.^15^

To date, few studies have examined the clinical and neurobiological differences between nonresistant and resistant MDD,^16^ although risk factors for resistance, including high recurrence rates (up to 80%),^17^ failure of initial AD, psychiatric comorbidity,^18^ undetected hypomanic symptoms,^9^ increased cardiac morbidity,^19^ and mortality,^17^ have been identified. There are also preliminary data from small studies to suggest TRD is associated with biological determinants, including hyperactivity in the anterior cingulate cortex, striatum and amygdala,^20^ altered neurotransmitter levels,^21^,^22^ and genetic polymorphisms.^23^–^25^

**Clinical Implications**

- The high level of TRD in primary care highlights the necessity to provide ongoing psychiatric education for physicians.
- Treatment of TRD should also address medical comorbidities, including chronic pain and obesity, which could exacerbate depressive symptoms.
- Standardized assessments should be incorporated into the clinical interview to more objectively assess AD outcome.

**Limitations**

- The sample was not selected at random.
- AD compliance was based on patient self-report.
- The retrospective design precluded longitudinal follow-up.

The large population of patients with MDD seen in primary care provides an opportunity to estimate TRD prevalence and its associated characteristics. While it may be assumed that the presentation of MDD in primary care clinics is not as severe or chronic, several investigations have reported a lack of demographic or symptom differences between primary care and tertiary samples of MDD.^26^,^27^ Further, studies^28^–^31^ have shown that at least 10% of primary care visits are related to depression: 1 group demonstrated that during the year of an index episode of depression, 90% of patients who participated in the Canadian National Population Health Survey visited their GP at least once, and one-third had at least 6 visits to their primary care physician, indicating a high level of contact.^32^ This high presence of depression in primary care may be due to several factors, including ease of access to a GP, compared with a specialist, lack of specialists in a patient’s vicinity, or lengthy wait-list times to see a specialist. Indeed, effective MDD treatment can be offered through primary care if interventions are evidence-based.^33^ In addition, evaluating TRD in primary care provides an opportunity to assess how patients are managed, thereby highlighting whether there is a need for increased dissemination of guidelines to ensure appropriate care.

The aim of the InSight study was 3-fold: to assess the prevalence of TRD in primary care; to extend existing research about the differences in clinical characteristics of TRD, compared to nonresistant, patients; and to assess how patients with TRD are managed in primary care.

## Methods

### Subjects and Study Criteria

This was a multicentre, retrospective chart review of patients, aged 18 to 75, with a documented primary diagnosis of MDD, based on physician report of the Diagnostic and Statistical Manual of Mental Disorders, Fourth Edition, criteria, who were receiving AD treatment from their primary care physician. Patients with psychiatric or medical comorbidity were not excluded from the study. Patients in this study were considered treatment-resistant if they were observed by the treating physician to have no or minimal improvement following 2 or more AD trials that were a minimum of 6 weeks in duration. While cognitive-behavioural therapy would have been an acceptable treatment alternative, the lack of consistent availability across sites precluded its inclusion as a treatment option.

A total of 135 primary care physicians from across Canada agreed to participate in the InSight Registry: British Columbia (*n* = 34), Alberta (*n* = 10), Saskatchewan (*n* = 1), Manitoba (*n* = 4), Ontario (*n* = 39), Quebec (*n* = 31), and the Atlantic provinces (*n* = 16). Physicians were contacted based on a registry of primary care physicians through the CHRC. Each physician was asked to review the charts from 10 consecutive patients meeting criteria for MDD, and to complete the CRFs, which were sent via facsimile to the InSight data coordinating centre.

This study was approved by the central Research Ethics Board in each province. Data collection took place between October 2008 and August 2009.

### Procedures

The data collection form was developed to capture information regarding sex, age, employment status, BMI, vital signs, medication history, and side effects associated with current medication regimen. Depression severity, and the scale used for its evaluation, were recorded, if completed. Questions to the primary care physician regarding future management were also included: “When will you assess if you have to change treatment?” (reported in weeks); “How will you decide when treatment needs to be altered” (question patient, use a formal scale, wait until patient complains about no response or side effects occur); “How might you alter the treatment?” (refer to psychiatrist or psychologist, refer to other mental health care provider, increase dose of AD, combine with another agent, switch to another agent, do nothing further); “If combining medications, which agent would you use?” (any psychotropic captured); and “If switching meds, which agent would you use?” (any psychotropic captured).

CRFs were scanned using an optical recognition software program (Cardiff TeleForm, New England Survey Systems, Brookline, MA). The data were stored in an electronic database at the CHRC.

### Statistical Analysis

Continuous variables are summarized as median and interquartile range. Discrete variables are reported as counts and percentages. For continuous variables, differences between TRD and non-TRD patients were compared using the Kruskal–Wallis test. Categorical variables were tested using Pearson chi-square or Fisher exact tests if the number of patients was fewer than 5. Analyses were performed using SAS software version 9.2 (SAS Institute Inc, Cary, NC) and tested using 2-sided tests at a significance level of 5%.

## Results

### Treatment-Resistant Depression Prevalence

**Figure 1:**
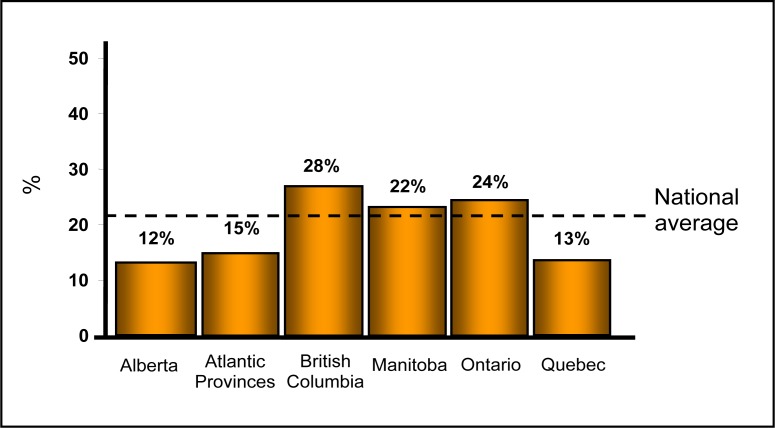
**Prevalence of treatment-resistant depression across Canada**

A total of 1282 charts of treatment-seeking depressed patients from 135 physicians were reviewed. Complete data were available for 1212 patients, which formed the full analysis set. Among these patients, 263 were classified as TRD, resulting in a prevalence of 21.7% across Canada (Figure 1). The prevalence across provinces varied (*P* < 0.001), with British Columbia having the highest rate (28.7%) and Alberta the lowest rate (12.8%). Owing to the small sample size, the TRD prevalence in Saskatchewan was not included (2 patients with TRD in a sample of 4).

### Treatment-Resistant Depression Characteristics

Sex and ethnicity did not differ between non-TRD and TRD groups (Table 1). However, higher age (50, compared with 47, years, *P* = 0.01), duration of current episode (36 months, compared with 12 months, *P* < 0.001), and greater work impairment were observed in TRD, compared with non-TRD, patients, with 17.6% of patients with TRD on long-term disability, compared with 10.1% in the non-TRD group (*P* = 0.002).

Axes I, II, and III comorbidities were also higher in the TRD, compared with the non-TRD, group (online eFigure 2). Axis I comorbidity in the TRD group was primarily accounted for by anxiety and substance use disorders. The most prevalent Axis II disorder was in the Cluster C category (anxious–fearful) in the TRD, compared with the non-TRD, group (35.0% and 23.6%, respectively, *P* < 0.001), followed by Cluster B and A disorders in smaller proportions. Among Axis III comorbidities, cardiovascular disease (21.3%), chronic pain (21.7%), primary insomnia (16.4%), and type II diabetes (14.8%) were the most prevalent (Table 2). Further analyses regarding weight revealed that patients with TRD had a higher BMI (28.3 kg/m^2^ and 26.3 kg/m^2^, respectively, *P* < 0.001), and obesity (BMI > 30 kg/m^2^) was associated with treatment resistance (60.1% and 44.8%, respectively, *P* < 0.001). Patients with TRD were also more likely to be on lipid-lowering, hypoglycemic and (or) insulin, or antiinflammatory drugs.

**Figure 3:**
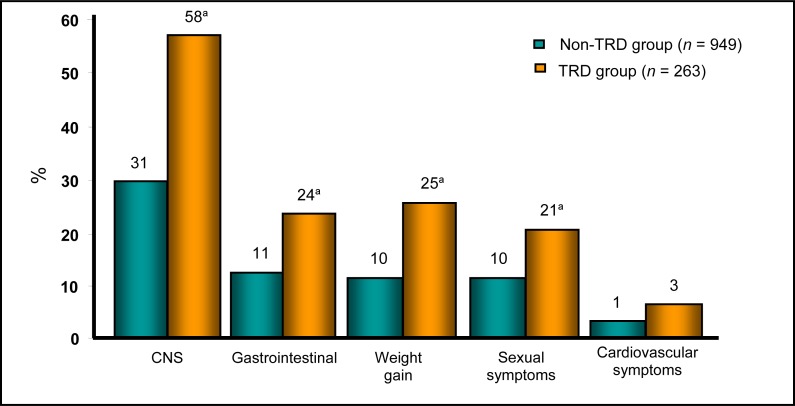
**Percentage of side effects reported** CNS = central nervous system; TRD = treatment-resistant depression ^a^ *P* < 0.05

Patients with TRD also reported a greater number of side effects attributed to their current AD regimen: central nervous sysem (for example, drowsiness, dizziness, dry mouth, confusion, headache, tremors, and blurred vision), gastrointestinal (for example, nausea, constipation, and diarrhea), cardiovascular (for example, rapid heart beat), as well as weight gain and sexual dysfunction across the domains of desire, arousal, and orgasm (Figure 3).

### Depression Management

#### Diagnosis and Screening

Patients with TRD, compared with no TRD, were more likely to have ever been referred to a psychiatrist (71.7% and 31%, respectively, *P* < 0.001). They were also more likely than non-TRD patients to have been questioned about BD symptoms (52.1% and 40.1%, respectively, *P* < 0.001), although this only occurred in about one-half of all patients.

Only 25% of physicians indicated that they conducted standardized assessments of depression severity (23.9% in the non-TRD group and 31.1% in the TRD group, nonsignificant). The scales used were the HAMD-7,^34^ the Patient Health Questionnaire-9,^35^ the Montgomery– Åsberg Depression Rating Scale,^36^ or the Beck Depression Inventory,^37^ with the HAMD-7 being the most frequently used.

##### Psychotropics.

Overall, venlafaxine XR, citalopram, bupropion XL, and escitalopram were the most frequently prescribed (> 10% of sample in each case) ADs (23.8%, 19.3%, 17.7%, and 12.8%, respectively), with minimal use of tricyclic ADs or monoamine oxidase inhibitors. Bupropion XL was more often prescribed for patients with TRD (23.6% and 16.1%, respectively, *P* = 0.005). Dosing across the most frequently used ADs was only higher for venlafaxine XR and bupropion in the TRD group (Table 3). The TRD group was also more likely to be prescribed at least 2 psychotropics (57% and 25.1%, respectively, *P* < 0.001), as well as atypical APs (19.8% and 5.6%, respectively, *P* < 0.001) and benzodiazepines (15.6% and 8.0%, respectively, *P* = 0.002), compared with the non-TRD group.

##### Subsequent Treatment Strategies.

There were no between-group differences for the duration physicians waited to assess if a change in treatment was necessary (8 and 6 weeks, respectively, *P* = 0.78). The decision to change treatment was more often based on reported lack of effectiveness than side effects. The intervention differed between groups, where patients with TRD were more likely to be referred to a psychiatrist or psychologist (50.2% and 34.5%, respectively, *P* < 0.001), and non-TRD patients were more likely to have their dose increased (61.0% and 52.5%, *P* = 0.01). There were no between-group differences in whether a patient was offered a combination treatment or switched to another AD. When combining medications, patients with TRD were most likely to be prescribed an atypical AP, while non-TRD patients were more likely to receive bupropion XL, adjunctively. When switching medications, venlafaxine XR, escitalopram, bupropion XL, and duloxetine were most frequently selected (28.4%, 14.4%, 13.8%, and 12.6%, respectively). Non-TRD patients were more likely to be switched to venlafaxine XR, with no other group differences across escitalopram, bupropion, and duloxetine.

## Discussion

### Treatment-Resistant Depression Prevalence

In our study, the Canada-wide prevalence of TRD in primary care was 21.7%, compared with a rate of 30% in the STAR*D trial,^38^ where patients were recruited both from specialty clinics and primary care settings. These findings emphasize the persistence of depressive symptoms in a large proportion of patients with MDD, despite adequate trials of at least 2 ADs.

### Treatment-Resistant Depression Clinical Characteristics

Published reports indicate TRD is associated with early age at onset, a more complex illness course (for example, high frequency of prior episodes), as well as psychiatric and medical comorbidity (for example, anxiety or personality disorders, and cardiac diseases).^18^,^39^ While we did not capture episode recurrence and age of onset, our findings are consistent with differences in age, duration of episode, and cardiovascular disease. We also report increased presence of additional comorbidities, medication use, side effects, and decreased work function in patients with TRD.

Over 50% of TRD patients had an Axis I or III diagnosis, and over 30% had a comorbid personality disorder. Consequently, for a subgroup of depressed patients with comorbidities, existing medications may be inadequate to relieve the additional symptoms. Importantly, comorbidity contributes to a higher all-cause mortality rate for patients with TRD, which is reported to be about 13% during 4 to 8 years^40^ and 32% during 7 years.^41^ These findings also emphasize the importance of different treatment strategies to target multiple psychiatric and medical conditions. However, the resulting increase in medication use may also play a role in decreased AD efficacy through drug interactions, as well as increased side effect burden. The higher number of adverse events in the patients with TRD observed in this study may be due to an increase in medication use, compared with the non-TRD group. In addition, considering this was a cross-sectional study, it is also difficult to disambiguate treatment-emergent adverse events and worsening depression symptoms.

Notably, patients with TRD had higher BMI and presence of obesity, which is reflective of the increased rates of cardiovascular disease and diabetes in MDD, and potentially in TRD.^19^ Additionally, increased use of atypical APs can also lead to increased body weight.^42^ One proposed consequence of increased body weight is reduced AD efficacy, especially where BMIs exceed 25 kg/m^2^.^43^–^45^ Several explanations have been suggested for this change in AD efficacy with higher BMI, including increased inflammatory activity, effects on the hypothalamic–pituitary–adrenal axis, increased neurovegetative symptoms, such as disturbed sleep and appetite, as well as pharmacokinetic alterations that occur with greater body fat, resulting in reduced drug bioavailability.^45^–^47^

The negative impact of depression on work function (for example, presenteeism, absenteeism, unemployment, and long-term disability) is well documented.^48^–^51^ However, the effects of TRD on work status is less clear. In our study, we report a higher rate of long-term disability in the TRD group, which contributes to the increased economic burden in this subpopulation. While employed non-TRD patients missed more work than employed patients with TRD, this may be driven by the finding that non-TRD patients are more likely to remain employed, and consequently miss more work days.

### Depression Management

It is important to note that there can be several barriers to effective physician treatment of depressed patients, including physician attitudes and reluctance to initiate therapy, which should be further evaluated.^52^,^53^ However, clinical practice guidelines are also a useful tool to aid psychiatric management. According to current treatment guidelines, psychiatric management should begin with a thorough evaluation of symptoms and their impact on function, with an emphasis on suicidality, followed by a clarification of polarity to rule out BD, as well as additional details on comorbidity and concomitant medications.^54^ In our study, only 50% of patients are screened for bipolarity. Importantly, a significant proportion of patients who were unresponsive to ADs may have undetected BD.^55^,^56^ Further, the use of validated depression scales should be used to monitor patient progress, which will help to determine improvement (more than a 20% decrease in depression scores after 2 weeks), response (more than a 50% decrease in depression scores after adequate treatment duration), as well as to identify treatment-emergent adverse effects and unresolved symptoms that would warrant other treatment strategies.^57^ As only 25% of clinicians used a formal scale in our study to evaluate symptoms and the decision to change treatment was primarily driven by patient questioning, further education on the benefits of tracking symptoms quantitatively is warranted.

Regarding treatment, primary care physicians tended to follow the prevailing guidelines. While pharmacotherapy was the mainstay for treatment, referral for neurostimulation therapies for TRD patients, in particular, would be indicated.^57^ All of the commonly prescribed medications reported (that is, bupropion XL, citalopram, escitalopram, and venlafaxine XR) were considered first-line agents at the time of the study. However, the duration of time before treatment adjustment was longer than recommended: 6 weeks for the TRD group and 8 weeks for the non-TRD group. Current evidence suggests making a change if there is no improvement after 4 to 6 weeks.^58^ This is due to findings that lack of early improvement is predictive of nonresponse.^59^,^60^

Switching to an agent with demonstrated superiority (for example, duloxetine, escitalopram, sertraline, or venlafaxine XR) or prescribing adjunctive therapy (for example, aripiprazole, olanzapine, risperidone, or lithium) are considered first-line recommendations for nonresponse.^58^ In our study, while the switch strategies adhered to the guidelines, it should be noted that switching to bupropion XL is considered a second-line treatment strategy, owing to lack of evidence of superiority over other agents, as well as increased risk for drug interactions.^58^ However, treatment should be individualized based on symptom profile, in addition to other factors, including tolerability, comorbidity, and previous response. Therefore, in cases where patients are having difficulty with sexual side effects, for example, bupropion would be an appropriate medication.^61^ The use of atypical APs was more likely to be used in the TRD group, which is also consistent with MDD guidelines. However, a greater proportion of patients with TRD received a benzodiazepine, compared with non-TRD patients. While duration of use was not specified, there is a consensus across guidelines that benzodiazepines should only be used in the short term (no more than 4 weeks), owing to the lack of efficacy data beyond this time point,^62^,^63^ as well as the increased risk of abuse, cognitive impairment, and falls in the elderly.^58^,^64^,^65^

A primary limitation of our study is the retrospective data collection method. It should be noted that this may not be a representative sample of primary care patients, owing to a lack of random sampling and potential selection biases. Other limitations include physician report of psychiatric and medical diagnoses instead of a structured interview, no correction for multiple comparisons, and no accounting for any strata or clustering of physicians across sites. In addition, it is generally accepted that TRD reflects a failure of at least 2 adequate AD trials; however, treatment compliance was not assessed. It is also unclear whether true treatment resistance may require further nonresponse.

Nevertheless, this report provides a valuable snapshot of physician-reported management of one of the most prevalent psychiatric disorders encountered by primary care physicians in Canada. Further longitudinal characterization of TRD is necessary, and future research should examine the prevalence of TRD in different settings and evaluate biomarkers that could aid in treatment selection.

In summary, TRD is prevalent, posing a significant issue, owing to its association with functional and symptom burden. The management of patients within a primary care sample from across Canada mostly followed clinical guidelines regarding AD choice, duration, and treatment strategies. However, further dissemination of recommended guidelines, including earlier treatment adjustments and training on the use of formal scales, would improve current treatment practices.

## 



## Figures and Tables

**Table 1:** Patient demographics

Variable	Non-TRD group *n* = 949	TRD group *n* = 263	*P*
Sex, *n* (%)			0.65
Male	303 (32.3)	81 (30.8)	
Female	635 (67.7)	182 (69.2)	
Age, years^a^	47 (37–57)	50 (42–58)	0.01
Ethnicity, *n* (%)			0.34
Caucasian	846 (90.0)	240 (91.9)	
Others	94 (10.0)	21 (8.1)	
Working status, *n* (%)			0.002
On disability	92 (10.1)	44 (17.6)	
Unemployed	161 (17.8)	46 (18.4)	
In school or homemaker	178 (19.6)	49 (19.6)	
Employed	476 (52.5)	111 (44.4)	
Among those employed			0.02
Missed work	174/473 (36.8)	26/106 (24.5)	
Did not miss work	299/473 (63.2)	80/106 (75.5)	
Duration of current episode, months^a^	12 (6–36)	36 (12–108)	<0.001

TRD = treatment-resistant depression

a Median, interquartile range

**Table 2:** Axis III comorbidities screened

Variable	Non-TRD group *n* = 949	TRD group *n* = 263	
	*n* (%)	*n* (%)	*P*
Cardiovascular disease	151 (15.9)	56 (21.3)	0.04
Chronic pain	137 (14.4)	57 (21.7)	0.005
Sleep disorder	74 (7.8)	43 (16.4)	<0.001
Type II diabetes	88 (9.3)	39 (14.8)	0.009
Arthritis	96 (10.1)	32 (12.2)	0.34
Asthma	68 (7.2)	22 (8.4)	0.51
Chronic obstructive pulmonary disease	22 (2.3)	15 (5.7)	0.005
Cancer	29 (3.1)	13 (4.9)	0.14
Osteoporosis	38 (4.0)	10 (3.8)	0.88
Chronic kidney disease	18 (1.9)	7 (2.7)	0.44
Hepatitis	5 (0.5)	5 (1.9)	0.045
Peripheral vascular disease	12 (1.3)	4 (1.5)	0.76

TRD = treatment-resistant depression

**Table 3:** Dosing of current therapy

Variable	Non-TRD group *n* = 949	TRD group *n* = 26	*P*
Number of current psychiatric medications, *n* (%)			<0.001
1	711 (74.9)	113 (43.0)	
2	238 (25.1)	150 (57.0)	
Total daily dose, median (interquartile range)			
Venlafaxine XR	150 (75–225)	187.5 (75.0–262.5)	0.02
Citalopram	20 (20–40)	30 (20–40)	0.37
Bipropion XL	150 (150–300)	300 (150–300)	0.002
Escitalopram	10 (10–20)	10 (10–20)	0.21

TRD = treatment-resistant depression; XL = long acting; XR = extended release

## Abbreviations

AD: antidepressant
AP: antipsychotic
BD: bipolar disorder
BMI: body mass index
CRF: case report form
CHRC: Canadian Heart Research Centre
GP: general practitioner
HAMD-7: Hamilton Depression Rating Scale—7 item
MDD: major depressive disorder
STAR*D: Sequenced Treatment Alternatives to Relieve Depression
TRD: treatment-resistant depression
XL: long acting
XR: extended release
