# Supplementary figures and images for: Treatment-Resistant Depression in Primary Care Across Canada
Source: Can J Psychiatry. 2014 Jul;59(7):349–57. doi: 10.1177/070674371405900702 (PMC4086317; doi:10.1177/070674371405900702)

**Figure 2: Comorbid Conditions**

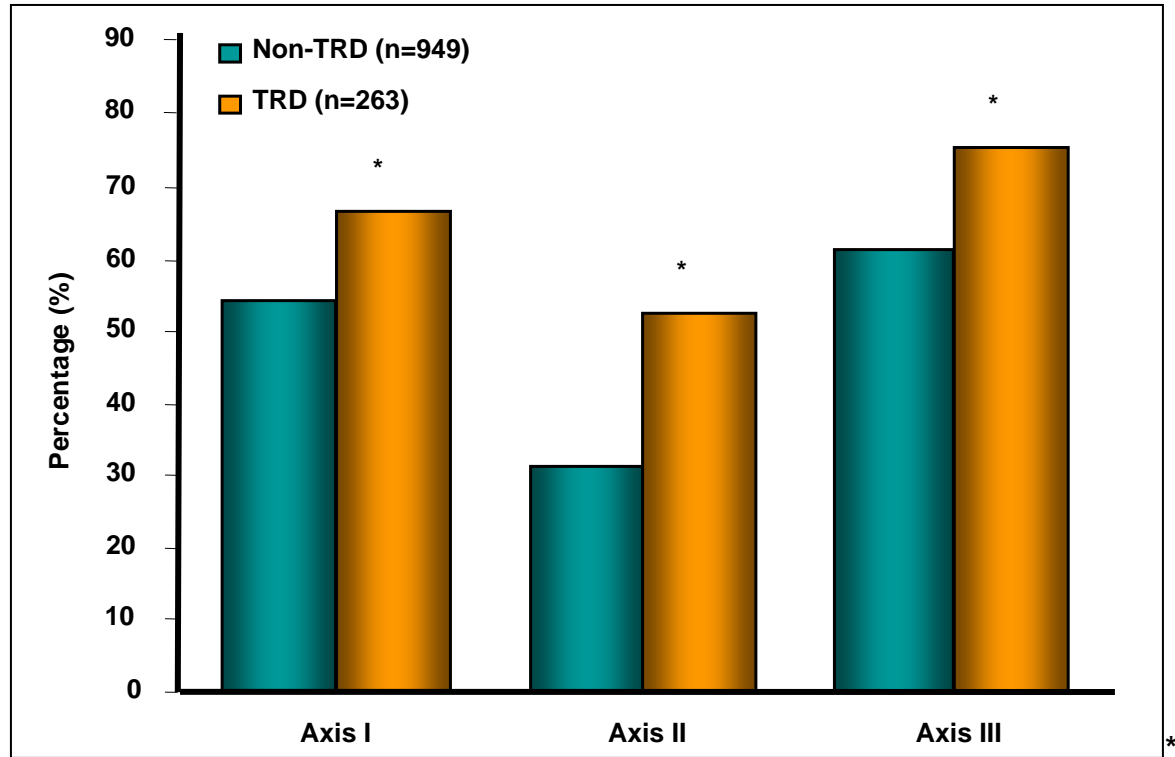

p<.05

Supplement: Supplementary file 1 [file CJP-2014-vol59-July-349-357-eFig2.pdf]
